# 

**Published:** 1884-06

**Authors:** 


CONTENTS.

| | Page. |
|---|---|
| Indigestion | 99 |
| Prevention of Disease | 100 |
| Heart Disease | 101 |
| Cleanliness | 103 |
| Food for Brain and Nerves | 105 |
| Starvation versus Developement | 106 |
| An Article for Every Family | 106 |
| An Antidote for Hydrophobia | 107 |
| Carpet Beating | 108 |
| Thunder and Lightning | 109 |
| Eating Crow | 111 |
| Early Rising | 112 |
| The Good Old Times | 113 |
| Errors in Domestic Medicine | 113 |
| Sneezing and Shivering | 118 |

